# Bevacizumab Plays a double-edged role in Neoadjuvant Therapy for Non-metastatic Breast Cancer: A Systemic Review and Meta-Analysis

**DOI:** 10.7150/jca.53303

**Published:** 2021-03-05

**Authors:** XinJie Chen, Yu Gao, GanLin Zhang, BingXue Li, TingTing Ma, YunFei Ma, XiaoMin Wang

**Affiliations:** 1 Beijing University of Chinese Medicine, No. 11, Bei San Huan Dong Lu, Chaoyang District, Beijing, 100029, China.; 2 Oncology Department, Beijing Hospital of Traditional Chinese Medicine, Capital Medical University, No.23 Back Road of Art Gallery, Dongcheng District, Beijing, 100010, China.

**Keywords:** Bevacizumab, neoadjuvant therapy, surrogate endpoint, prognostic biomarker, non-metastatic breast cancer.

## Abstract

The anti-angiogenic drug Bevacizumab (Bev) is engaged in neoadjuvant therapy for non-metastatic breast cancer (NMBC). However, whether neoadjuvant Bev providing a greater benefit to patients is debatable. Our study aimed to review Bev's role in Neoadjuvant therapy (NAT) in NMBC and identify predictive markers associated with its efficacy by systemic review and meta-analysis. Eligible trials were retrieved from the Pubmed, Embase, and Cochrane Library, and random or fixed effects models were applied to synthesize data. Power of pCR to predict DFS or OS was evaluated by nonlinear mixed effect model. In NMBC, Bev significantly improved the rate of patients achieving pCR, but this benefit discontinued in DFS or OS. Biomarkers such as PAM50 intrinsic subtype, VEGF overexpression, regulation of VEGF signaling pathway, hypoxia-related genes, BRCA1/2 mutation, P53 mutation and immune phenotype can be used to predict Bev-inducing pCR and/or DFS/OS. Unfortunately, although patients with pCR survived longer than those without pCR when ignoring the use of Bev, but patients achieving pCR with Bev might survive shorter than those achieving pCR without Bev. Subgroup analyses found Bev prolonged patients' OS when given pre- and post-surgery. Lastly, adding Bev increased adverse effects. Overall, Bev offered limited effect for patients with NMBC in an unscreened population. However, in biomarkers - identified subgroup, Bev could be promising to ameliorate the prognosis of specific patients with NMBC.

## Introduction

Breast cancer (BC) is the most frequently diagnosed cancer and the leading cause of cancer-related death among women 1. With the popularization of self-test and improvement of screening, an early diagnosis is possible 2. However, there are still appreciable number of patients with stage I-III BC suffer from recurrence and metastasis, which leads to reduced survival time 3. Therefore, it is particularly critical to find appropriate early intervention to prevent or delay the recurrence or metastasis.

Neoadjuvant therapy (NAT) is usually used to shrink tumors to allow surgery to be performed and increase proportion of patients to receive breast-conserving surgery (BCS). Generally, patients obtained pathological complete response (pCR) from NAT have improved survival 4, making it a decent approach of curing primary non-metastatic breast cancer (NMBC). Meanwhile, angiogenesis plays a key role in the growth and metastasis of BC 5-7. VEGF, which is a powerful inducer of angiogenesis and a predictive marker of poor prognosis for cancer 8, 9, has been considered a predominant target in controlling cancer progression.

Bevacizumab (Avastin; Genentech) is a humanized anti-VEGF-A monoclonal antibody to inhibit angiogenesis and subsequently suppress tumor growth and metastasis 10. Thereby, bring bevacizumab into NAT to treat NMBC seems to be a feasible strategy. However, clinical application of Bev is a controversary topic. Although most clinical trials using Bev in NAT reported improved pCR, few of them achieved improved disease-free survival (DFS) or overall survival (OS). Even worse, reduced DFS was observed in patients achieving pCR in the study of Wan G et al. 11. Similar meta-analysis of Bev-containing NAT have been published, but some had adjuvant rather than neoadjuvant treatment trials admixed 12, 13 and other paid inadequate attention to the inconformity between pCR and survival outcomes 14. Whether NMBC patients benefit from Bev-containing NAT or which group of them benefit the most remains unknown.

The purpose of our study was to review Bev's role in NAT of NMBC and identify specific subgroups that may gain advantage from Bev-containing NAT. Here we performed a systematic review and meta-analysis to further evaluate the impact of Bev on DFS/OS in NAT of NMBC.

## Materials and Methods

### Protocol and registration

We conducted and reported this systemic review and meta-analysis following the PRISMA 2009 checklist 15. Our study was registered on PROSPERO website with identifier CRD42020175912.

### Search strategy

Pubmed, Embase and Cochrane Central Register of Controlled Trials were searched to identify eligible studies published before March 31th/2020. Search strategy was generated using PICOS principle. P (population-patients with breast cancer), I (intervention-neoadjuvant bevacizumab), S (study-randomized controlled trial, RCT) were restricted. C (comparator) differs along with different studies and O (outcome) was not limited. For included studies, manual retrieval was executed to supplement results. Medical terms used in search strategy were listed in **Appendix A**.

### Eligibility criteria

Only RCTs of BC were considered to filter by the following criteria; 1) Patients had primary NMBC. 2) Patients in one arm received Bev-containing intervention prior to surgery while patients in other one arm applied the same treatment schedule but without Bev or with placebo. 3) Reported the outcome. 4) Patients took Bev for more than 3 months. Trials were excluded if 1) Bev was not the experimental drugs; 2) metastatic cases were included; 3) Bev was applied only in postoperative adjuvant therapy; 4) several studies were conducted in same population, only 1 of them would be included; 5) data on any outcome was not available.

### Study selection and quality assessment

Literature was screened and assessed risk of bias by two researchers independently. For cases of any disagreement, they discussed with third opinion until consensus. Quality assessment was performed for studies in meta-analysis, referring to Cochrane risk-of-bias assessment tool.

### Data extraction

Information about trial name, country, year of registration, sample size, PICO, follow-up time and all interested outcomes was extracted independently by two authors. Nun-metastatic breast cancer was defined as breast cancer without distant metastasis. pCR was defined as absence of invasive cancer in the breast and lymph nodes (ypT0/TisN0) and bpCR was defined as absence of invasive cancer in the breast (ypT0/TisNx). Data of every outcome was extracted along with the number of evaluated participants and events. Of note, event free survival (EFS) was regarded as same as DFS. Surgery outcomes were categorized as either lumpectomy (BCS) or mastectomy for the convenience of statistics. For toxicity outcomes, grade 3-4 adverse events (AEs), assessed by National Cancer Institute's Common Terminology Criteria for Adverse Events (NCI CTCAE), version 3.0 or 4.0, were extracted.

### Statistical analyses

Cochrane's Q test and Higgins I-squared statistic were utilized to detect the heterogeneity of the included studies. High heterogeneity was defined as p for Q test <0.1 or I2 >50%. Random-effect model and Der-Simonain-Laird method were used for cases with high heterogeneity. Otherwise, fixed-effect model and Inverse-variance method were undertaken. Funnel plot was drawn to observe and exhibit the publication bias. Statistical significance was defined as a 2-side p<0.05. All statistical analyses were taken by Stata 16.0.

To estimate the association between pCR OR and DFS/OS HR at trial-level, a nonlinear mixed-effects model was fitted 16. It provided several parameters considered measurement error (OR or HR with their 95% confidence interval (CI)) to paint linear-like relationship by regarding pCR OR as independent variable and DFS/OS HR as dependent variable. If pCR was a good surrogate for survival outcomes in a series of trials, a larger pCR OR would lead to a smaller DFS/OS HR and β which represents slope should be negative, and vice versa. Calculation of parameters was performed in R software with the R source package developed by Korn et al. 16. (Available from https://brb.nci.nih.gov/programdownload/pCRsoftware.html, last accessed on June 17th/2020.) β was considered statistically significant when estimate ± standard error didn't across 0.

## Result

### Study selection

**Figure 1:**
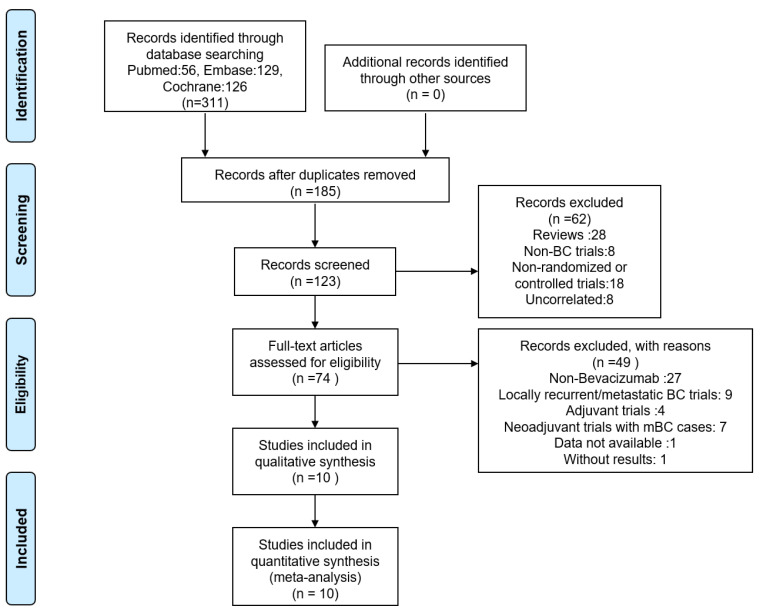
PRISMA Flow Diagram of Literature Screening Process.

A total of 311 literatures were retrieved and all of them were in English. After removing the duplicates, we decided 74 records related to RCTs of BC by reading titles and abstracts and finally identified 10 RCTs eligible by reading full-text articles and abstracts. Exclusion reasons by steps were listed in the PRISMA flow diagram, which was presented in **Figure 1**.

### Study characteristics

Most included studies were registered on clinicaltrials.gov between 2005 and 2011. Population involved was widely distributed, all at non-metastatic stage, mainly at stage II/III, no matter of human epidermal factor receptor 2 (HER2) or hormone receptor (HR) status. HER2-negative (HER2-) BC, which consists of triple negative BC (TNBC) and HER2-/HR+ BC, accounted largest number of patients. Various chemotherapeutic and few targeted agents were given with or without Bev. Among combinations of chemotherapy and Bev, taxanes combining with AC (doxorubicin+ cyclophosphamide) or EC (epirubicin+ cyclophosphamide) were the dominant prescriptions. All trials reported pCR (9 trials without ABCSG 32) or bpCR and half of them reported DFS and/or OS with median follow-up period ≥3 years. Essential characteristics for eligible studies were listed in **Table 1**.

### Risk of bias

**Figure 2:**
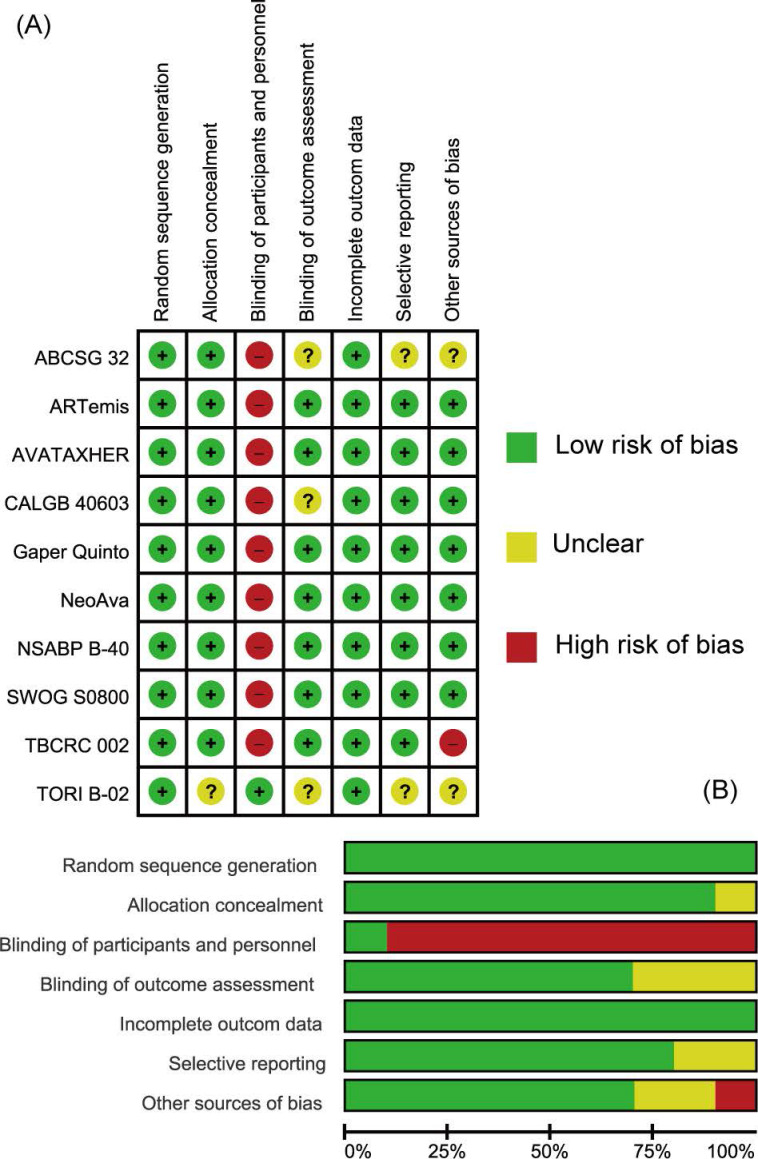
Assessment of Risk of Bias. (A) Summary of risk of bias. (B) Individual risk of bias. Green: Low risk of Bias; Yellow: Unclear; Red: High Risk of Bias.

The quality of individual studies assessed on the basis of pCR or bpCR outcomes by Cochrane Risk Bias Assessment Tool was summarized in **Figure 2**. Overall, included studies were well-designed RCTs. Most items were at low risk of bias especially those reported in the form of articles. All studies didn't apply blindness to participates and investigators except TORI B-02 trial. CALGB 40603 trial didn't give information about blinding of outcome assessment (mainly pCR) and TBCRC 002 trial didn't have patients' baseline characteristics balanced. ABCSG 32 and TORI B-02 trials, which were reported as abstracts, were assessed as Unclear in some items for their short of methodological information.

### Efficacy of Bev adding to NAT

**Figure 3:**
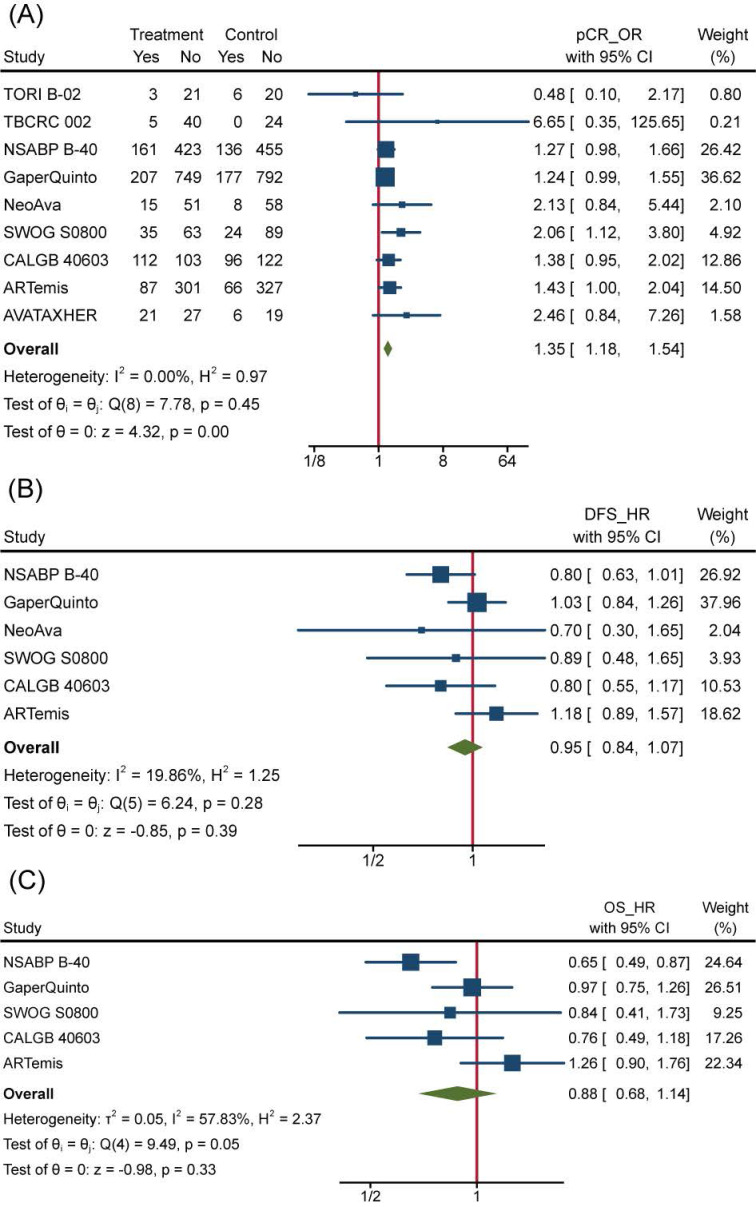
Efficacy of Bev Adding to NAT. (A)Pooled OR for pCR. (B) Pooled HR for DFS. (C) Pooled HR for OS.

To overview Bev's efficacy to NMBC when adding to NAT, we firstly performed data synthesis for effect-related outcomes. There is no doubt that Bev significantly improved pCR rate among 4849 patients (pts) involved in 9 trials (the other trial ABCSG 32 reported bpCR rather than pCR)**.** Pooled OR for pCR was 1.35(95%confidence interval (95%CI), 1.18~1.54) **(Figure 3A)**. Of 9 trials included, 8 showed improved OR for pCR and 2 of 8 reached statistical difference. We also performed meta-analysis for bpCR and objective response rate (complete response and partial response, ORR), discovering both of them showed similar improvement as pCR. OR was 1.34(95%CI, 1.17~1.54) for bpCR and was 1.77(95%CI, 1.46~2.13) for ORR **(Figure S1a and Figure S1b)**, which further confirmed Bev's short-term effect for patients with BC. Nevertheless, BCS rate after NAT across treatment arms kept close **(Figure S1c)**. OR for BCS in 3600 pts who received surgery in 5 trials was 1.02(95%CI, 0.88~1.18). Many factors would affect the decision on surgery type, whatever, the indiscriminate BCS rate provided a balanced starting line for subsequent follow-up across arms.

However, the improvement in pCR discontinued because incorporative HRs for DFS (pts=4695) and OS (pts=4565) are not significantly different **(Figure 3B and Figure 3C)**. All 6 trials included in the DFS analysis reported non-significant change as well as the synthetic effect, which was 0.95(95%CI, 0.84~1.07). 4 of 5 trials included in the OS analysis reported non-significant change while the leaving one got significant improvement of OS in Bev arm. As a result, pooled HR for OS was 0.88(95%CI, 0.68~1.14), meaning no improvement was observed on adding of Bev.

Certain transient efficacy and mediocre survival benefit aroused the controversy on 'Is Bev effective for NMBC on earth?'

To answer this question, it's important to evaluate: 1) if only part of patients of NMBC benefit from Bev; 2) if pCR is a reliable surrogate for DFS/OS.

### Biomarkers related to Bev's efficacy

Many biomarkers were identified to associate with pCR in included studies and some of them had the power to predict Bev-specific pCR. Here, we summarized these biomarkers that tested in tissue or serum at baseline to predict efficacy of adding Bev (**Table 2**). Patients with these molecular characteristics favored Bev-containing regimens in NAT in particular population with regard to given outcomes. As we expected, Bev's targeted therapeutic effect pronounced more in patients with high VEGF expression. Patients of HR+ BC with high VEGF-A had an increased chance of pCR and the interaction OR was 29.4 (p<0.001) 33. Other molecules related to angiogenesis, such as small RNAs regulating VEGF pathway and TMEM100 participating in vasculogenesis, also played roles in predicting Bev's effect. Furthermore, patients with a small hypoxia signature preferred Bev in NAT as hypoxic tumors are usually angiogenesis-dependent. Serum CAIX (sCAIX), which is also a marker of hypoxia, showed opposite effect: adding Bev improved pCR in patients with low sCAIX level while high sCAIX led to poor DFS underlying unknown mechanisms. Besides, BRCA mutation, p53 mutation, proliferation, low ER signaling in TNBC and immune gene signature in HER2-/ER+ BC were reported with the power to predict pCR. Specially, pCR is a strong predictor of DFS in patients without BRCA mutation, different from BRCA1/2 mutation carrier.

### pCR as surrogate of DFS/OS

**Figure 4:**
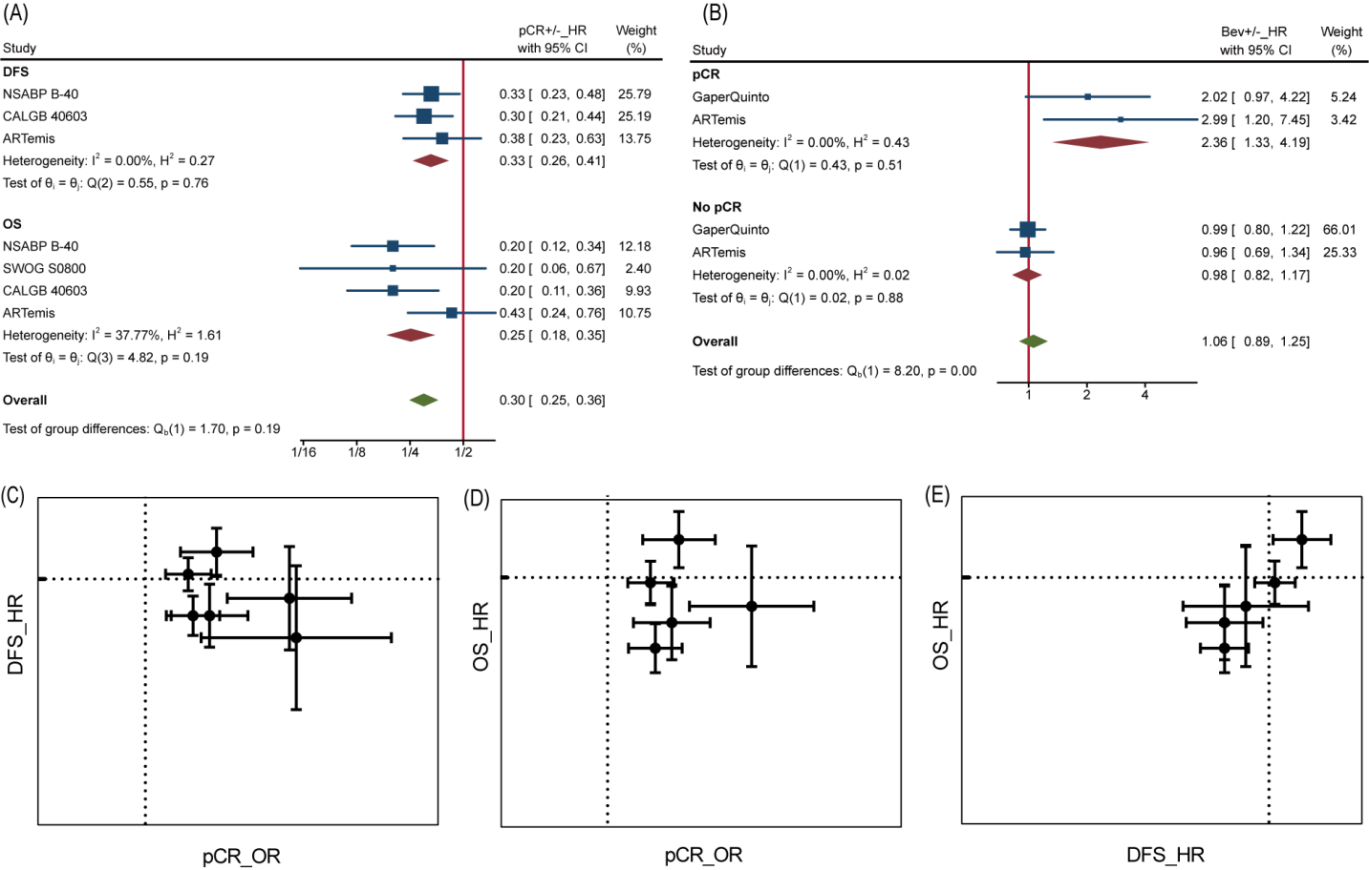
pCR as surrogate for DFS/OS. (A) HR comparing pCR and no pCR of DFS and OS. (B) HR comparing DFS among Bev arm and control arm in pCR and no pCR patients. (C) pCR as surrogate for DFS. (D) pCR as surrogate for OS. (E) DFS as surrogate for OS. In graph C-E, dotted line presents value 1.

Analyses at trial level showed poor relevance between pCR OR and DFS/OS HR. Neither DFS HR nor OS HR did appear to be dependent on pCR OR, from visualization of corresponding data (**Figure 4C and Figure 4D**). Likewise, the β values (slope) calculated by nonlinear mixed model 16, which took the measurement error of ORs and HRs into consideration, were far from being statistically significant (**Table S1**). Interestingly, a significant association was found between DFS HR and OS HR with β=1.59±1.01 (**Table S1**) and seen from the diagram (**Figure 4E**), demonstrating DFS was a good trial-level surrogate for OS in this NAT trials set.

It is reasonable that the implementation of pCR helps to achieve better survival, but perhaps this does not apply to Bev, as reported by Wan G et al. More in-depth analyses allowed us to confirm the inconsistency between pCR and DFS/OS. At individual level, pCR still was a powerful prognostic factor of DFS and OS as long as we ignore grouping. Patients with pCR were at one third risk of recurrence or metastasis compared to those without pCR for a HR of 0.33 (95%CI, 0.26~0.41) among 2423 pts, as well one fourth risk of death compared to those without pCR for a HR of 0.25 (95%CI, 0.18~0.35) among 2635 pts (**Figure 4A**). On the contrary, adding Bev played different prognostic roles in pCR and no pCR cases for DFS (**Figure 4B**). Among 449 pts with pCR, HR comparing Bev arm with control arm was 2.36 (95%CI, 1.33~4.19), indicating that Bev was associated with poor prognosis for patients achieved pCR. Among 2153 pts without pCR, the HR was 0.98 (95%CI, 0.82~1.17). Unfortunately, the trend continued in OS in same trials while data of Gaper Quinto trial was not available for synthesis. In summary, patients with pCR survived longer than those without pCR, but patients achieving pCR with Bev might survive shorter than those achieving pCR without Bev.

### Subgroup analyses of efficacy

**Figure 5:**
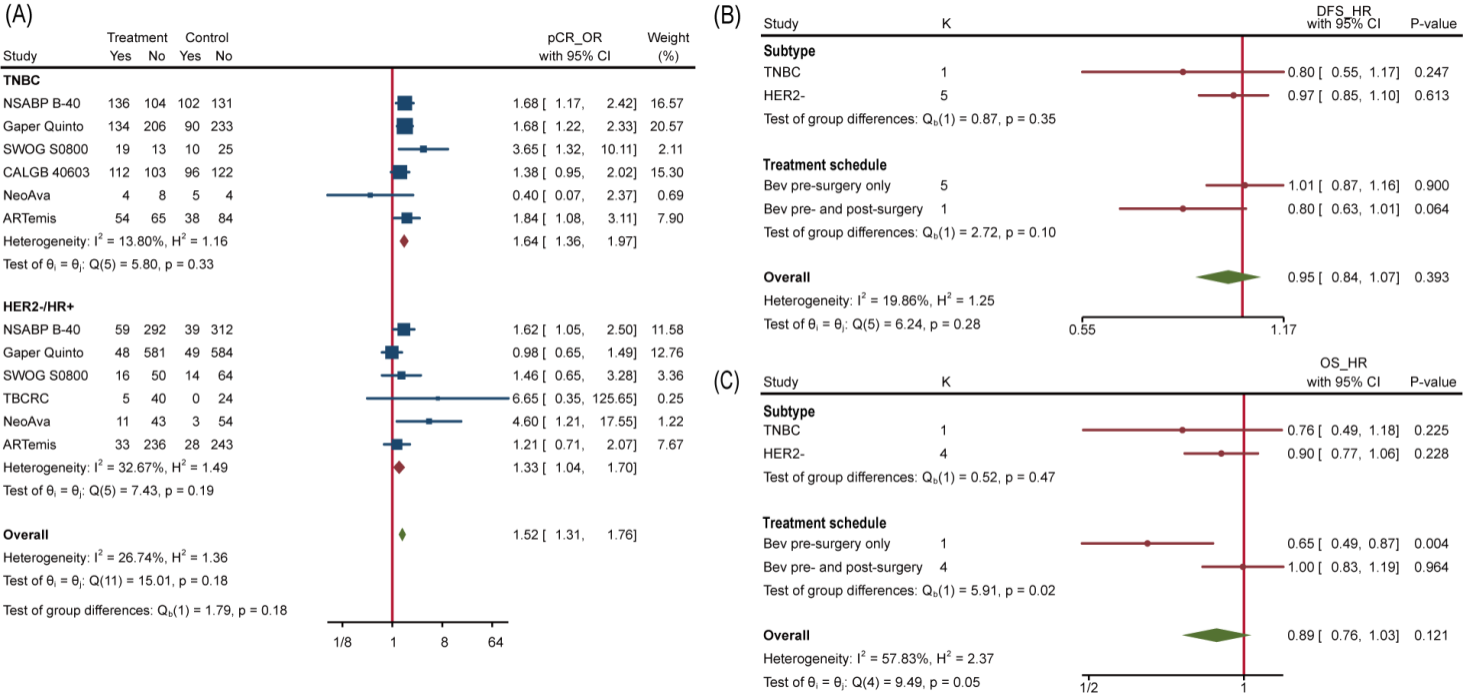
Subgroup Analysis. (A) Comparing pCR between TNBC and HER2-/HR+ BC. (B) Comparing DFS between different subtype and treatment schedule. (C) Comparing OS between different subtype and treatment schedule.

To explore which particular group of patients benefit more from Bev, we applied subgroup analyses. No interaction between subgroups of pCR was detected when studies were divided by HR status (HR-, HR+ or both), HER2 status (HER2+, HER2-) and total planned Bev dose (60mg/kg, 90/105mg/kg, 120mg/kg) (**Figure S2**). We further extracted separate data of TNBC and HER2-/HR+ BC to conduct subgroup analysis and the interaction test still showed no significance, with p=0.18 (**Figure 5A**). Similarly, none of included studies reported significant difference of pCR between TNBC and HER2-/HR+ BC. We then analyzed subgroups divided by treatment schedule (Bev pre-surgery only and Bev pre- and post-surgery) and disease subtype (HER2- BC and TNBC). For DFS, neither of them made it to distinguish populations with different prognosis (**Figure 5B**). Still, Bev pre- and post-surgery (1186 pts in 1 trial versus 3379 pts in 4 trials) earned better OS for patients in Bev arm, with p=0.02(**Figure 5C**), indicating that pre- and post-surgery medication of Bev may prolong patients' overall survival.

When it comes to PAM50 intrinsic subtype 40, the addition of Bev increased pCR rate in basal-like tumors but reducing pCR rate in non-basal-like tumors significantly with interaction p=0.02. Regrettably, age, tumor size, lymph node involvement and histopathological grade at baseline copied the situation of HR status, so none of them can separated a group that could benefit from Bev more.

### Adverse effects due to the addition of Bev

We analyzed word frequency of reported Grade 3-4 adverse events (AEs), and those appeared more than 3 times and overall occurrence rate higher than 1% were merged. Patients in Bev treatment group had more Grade 3-4 AEs and surgical complications than patients in control group. The most frequent AEs were hypertension and hand foot syndrome, both with p<0.01. In addition, neutropenia, febrile neutropenia, mucositis, headache also happened more in Bev-treated patients significantly. Overall, Bev rose patients' risk of experiencing AEs. We summarized the top AEs related to Bev across treatment arms in Table 3. Sometimes analogous items would be regarded as same item and the detailed data was summarized in **Appendix B**. In addition, Compliance evaluated by the proportion of patients completing all planed cycles was worse in Bev arm because of adverse effects (p<0.01).

### Publication bias

In the analysis of publication bias based on pCR, not a single plot fell outside the dotted line. Funnel plot didn't declare obvious publication bias in**Figure S3**.

## Discussion

Metastasis is a pivotal point in the prognosis of breast cancer and most breast cancer-related deaths are caused by metastasis. 5‐year relative survival rate of Stage IV breast cancer is only 26% although this index of whole BC patients has quite increased in past few decades 1. Hence, finding applicable intervention to prevent metastasis as well as recurrence is a core strategy for NMBC treatment. Our study demonstrated that Bev significantly improved the rate of patients achieving pCR, especially for those with specific biomarkers. PAM50 intrinsic subtype, VEGF overexpression, regulation of VEGF signaling pathway, hypoxia-related genes, BRCA1/2 mutation, P53 mutation, immune genes can be used to predict Bev-induced pCR. Of these, sCAIX level and BRCA1/2 mutation status were powered to predict Bev-related DFS. Nevertheless, no interaction was detected between age, tumor size, lymph node involvement, histopathological grade at baseline and Bev's efficacy. Note when patients were given Bev pre- and post-surgery in NSABP B-40 trial, not only OS but also distant metastasis free survival (not DFS) was prolonged significantly. Based on these evidences, the discordance between pCR and DFS/OS can be explained by wrong population and inadequate course of treatment.

Breast cancer exhibits highly spatial and temporal heterogeneity 43 as well as the clonal evolution of cancer cells 44, by which tumors' responses to drugs are deeply influenced. On one hand, Basal-like tumors are a subset of TNBC with more aggressive biological characteristics and poorer prognosis than luminal A tumors (ER- and/or PR-positive, and HER2-negative) 45, 46. With the addition of Bev, basal-like tumors achieved higher pCR rate while non-basal-like tumors got lower pCR rate 40.

Other biomarkers screened by our work, which was summarized in **Table 2**, can also contribute to identify Bev-sensitive subgroups. VEGF is the direct target of Bev, so it's reasonable that high serum VEGF, amplifications of VEGFA, VEGF pathway regulation RNAs are associated with Bev-inducing pCR. For example, serum VEGF-A level expanded pCR OR of HER2-/HR+ BC by 20 times. BRCA1 and BRCA2 are important DNA repair and tumor suppressor genes, whose deletion has been proved to promote TNBC cell proliferation 47. In the population classified by BRCA1/2 mutation, pCR became a strong predictor for DFS. On the other hand, time series analysis showed that amplifications of VEGF-A and TMEM100 sensitized tumor cells to the combination of Bev and chemo 36. Meanwhile, good responders displayed decrease of amplification-bearing subclones during medication while no responders didn't. With such knowledge, we get the chance to monitor patients' response to Bev and modulate treatment schedules as appropriate.

Besides, rapid growth caused by infinite proliferation puts tumors in hypoxic microenvironment. Hypoxia signature was linked to the response to Bev, but it's hard to tell that hypoxia leads to higher or lower pCR. What can be confirmed is that hypoxia induces vasculogenic mimicry (VM) 48 and the latter is vascular endothelial cell independent perfusion way. VM compensated the function of angiogenesis and represents greater invasiveness and poorer prognosis 49. VM, plus chemo-resist stem cells 50 and clonal evolution 44, makes Bev a double-edged sword in treating BC. In view of above cases, sifting before treatment and monitoring during treatment are vital for the application of Bev, not to mention the significantly increased adverse effects.

Immune gene signature also plays a role in Bev-inducing pCR. Immune system blends with every step of tumor progression, and cancer immune therapy have made drastically progress in past several years. As a highly immune infiltrated malignancy neoplasm 47, 51, breast cancer is promised beautiful landscape by immunotherapy 52. Immune gene signature is one of independent response predictors associating with Bev-containing regimen, making immune combining with vascular-targeted a hopeful therapy for BC. ECLIPSE trial (ClinicalTrials.gov Identifier: NCT03395899) is an example, in which Atezolizumab and Cobimetinib were given with Bev in an arm for women with untreated, histologically confirmed, operable, ER+, HER2-negative breast cancer. We are looking forward to the result of this trial.

When it comes to the discordance between the improvement of pCR and indifference survivorship, which initiated the analyses on efficiency of pCR as surrogate of DFS/OS, there is fog to be cut through in order to see the truth. It is a foregone conclusion that pCR implies better survival 4 for every single patient, especially when pCR is defined as the absence of invasive cancer cells and *in situ* residuals in the breast and lymph nodes (ypT0N0) 53. Our meta-analyses about HR comparing pCR and no pCR patients of DFS and OS declared this point (**Figure 4A**). Nevertheless, increase of pCR rate between treatment groups did not promise improved DFS or OS when we look at these trials vertically 4, 14. We inferred 3 points to comprehend the contradiction. First, unscreened population isn't suitable for Bev treatment. In fact, pCR has different power in predicting event-free survival (EFS) by BC subtypes, with HR ranging from 0.24(95%CI, 0.18~0.33) in TNBC to 0·63 (95%CI, 0.38-1.04) in HER2-/HR+ grade 1/2 BC 4. Comparing to whole BC population treated by Bev, patients with basal-like subtype or above-mentioned biomarkers seemed benefitting more from Bev-containing NAT. Second, pooled pCR OR is too small to translate to survival benefit. E. L. Korn et al. 16 investigated pCR's efficiency as a surrogate for early-stage Breast cancer at trial-level and advised a cut off value of 1.25 when take pCR as an intermediate screening end point as various confounding factors involved in long-term follow-up would eliminate drug's effect somehow. Using PAM50 intrinsic subtype and biomarkers to identify proper population for Bev targeted therapy at baseline can elevate the value of pCR OR and avoid the harmful effects to ultimately ineffective patients, thus making pCR a powerful surrogate for DFS/OS. Third, giving Bev medication only pre-surgery is not enough. Unique statistically significant HR was observed in NSABP B-40 trial, which is also the only trial where Bev was given pre- and post-surgery. If treatment schedule is the primary source of heterogeneity, perioperative Bev application would be a nice choice. Our surrogate analysis revealed no correlation between pCR OR and DFS/OS HR but significant correlation between DFS and OS in NAT trials. Whereas, same method declared no correlation between PFS HR and OS HR when giving Bev in first-line metastatic Breast Cancer (mBC) where Bev got PFS improved 54. It seems that Bev worked temporarily. As the conception of anti-angiogenic therapy is to induce tumor dormancy 55, existing evidence of angiogenesis-independent vascularization, cancer cells metabolic reprogramming and tumor microenvironment indicates that tumor can tolerate with blocked angiogenesis 56. With the withdrawal of Bev, cancer cells remaining *in situ* or hiding in the circulatory system might revive from dormancy 54, leading to recurrence or metastasis. However, the hypothesis needs to be verified modestly. This is because no invasive disease-free survival (IDFS) improvement was captured in adjuvant Bev-containing trials in early breast cancer 57, 58 although with high rate of early discontinuation and low event occurrence rate.

Last, adding Bev increased the frequency of grade 3-4 AEs and surgical complications significantly. Part of patients discontinued Bev because of toxicity ahead of schedule. In NSABP trial, only 289 patients of 587 (49.2%) completed all ten doses of Bev, the toxicity was partly responsible for it. Bev was generally given 15 mg/kg every three weeks (q21days) or 10mg/kg every two weeks (q14days), patients from NeoAva trial changed Bev dose from 15 mg/kg q21days to 10mg/kg every two weeks due to toxicity. The TORI B-02 trial set a group of 7.5 mg/kg q21days, of which patients achieved more pCR and less AEs numerically than the group of 15 mg/kg q21days with a small sample size. Besides, dose dense doxorubicin+ cyclophosphamide (ddAC) was scheduled in SWOG S0800 and CALGB 40603 trials. It was reported that metronomic regimens of cytotoxic anti-cancer drugs, called metronomic chemotherapy can be anti-angiogenic 59, 60. Antiangiogenic scheduling of chemotherapy combining with angiogenesis inhibitors in a continuous low-dose way can target tumor cells and endothelial cells at the same time with less toxicity. A Pilot Phase II Study combining metronomic chemotherapy and Bev of 10mg/kg q14days have been demonstrated to be effective and tolerable strategy for advanced non-squamous Non-Small Cell Lung Cancer. 61 Lately, a RCT study from China (ClinicalTrials.gov Identifier: NCT01112826.) revealed that 1 year of low-dose capecitabine maintenance therapy helped to improve TNBC patients' DFS. 62 To sum up, continuous low-dose of both Bev and toxic drugs could be an optimal method to manage toxic and side effects.

## Conclusion

In summary, Bev has a double-edged role in NAT of NMBC: on the good aspect, Bev improves patients' pCR and gains benefit for specific biomarkers sifted patients by prolonging their survival time; on the bad aspect, Bev worsens the prognosis for unscreened population even if they get to pCR, and produces AEs to interrupt treatment as well as lower the quality of life. Both hands require a prudent consideration on dose and course when applying Bev in clinical practice. With the development of individualized diagnosis and treatment, Bev can play the role of a candidate in the treatment of breast malignant neoplasm.

## Supplementary Material

Supplementary appendices, figures and table.

## Figures and Tables

**Table 1:** Essential Characteristics for Eligible Studies

Study	Country	Year*	Size	Population	Arms (number of patients)	Outcomes	Follow-up
TORI B-02 17	Ireland	2005	90	>3 cm, stage II/III, HER2- BC	DAC +Bev 7.5(n=28) DAC +Bev 15 (n=30) DAC + placebo(n=32)	pCR, Toxicity	NR
TBCRC 002 18	US	2005	75	Postmenopausal, stage II/III, HER2-/HR+ BC	Letrozole + Bev (n=50) Letrozole alone (n=25)	pCR, ORR, Toxicity, Surgery*	NR
NSABP B-40 19-21	US	2006	1206	T_1c-3_, N_0-2a_, M_0_, HER2- BC	D→AC(n=199)/D+ Bev→ AC+ Bev(n=195) DX→AC(n=204)/DX+ Bev→ AC+ Bev(n=196) DG→AC(n=191)/DG+ Bev→ AC+ Bev(n=201)	pCR, ORR, Toxicity, DFS, OS	4.7 years
Gaper Quinto 22-24	Germany	2007	1948	non-metastatic, HER2- BC, HR- or HR+ with positive nodes	EC→D (n=974) EC→D +Bev(n=974)	pCR, ORR, Surgery, Toxicity, DFS, OS	3.8 years
NeoAva 25, 26	Norway	2008	132	≥2.5cm, non-metastatic, HER2- BC	FEC→D/wP(n=66) FEC→ D/wP +Bev(n=66)	pCR, DFS	5 years
SWOG S0800 27	US	2009	215	stage IIB-IIIC, HER2- BC	nP +Bev→ ddAC(n=99) nP→ ddAC(n=63) ddAC→ nP(n=53)	pCR, EFS, OS, Safety	3 years
CALGB 40603 28-30	US	2009	454	stage II/III, TNBC	wP→ ddAC(n=115)/wP→ ddAC+ Bev(n=113) wPCarbo→ ddAC(n=113)/wPCarbo→ ddAC+ Bev(n=113)	pCR, Surgery, Safety, EFS, OS	3 years
ARTemis 31, 32	UK	2010	800	early-stage, HER2- BC	D→FEC(n=401) D→FEC+ Bev(n=399)	pCR, Surgery, Safety, DFS, OS	3.5 years
AVATAXHER 33	France	2010	73	early-stage, HER2+ BC, non-responders of chemotherapy	DT+ Bev(n=48) DT(n=25)	pCR, Surgery, Safety	NR
ABCSG 32 34	Australia	2011	100	early-stage, HER2+ BC	DT(n=25)/DT+ Bev (n=25) DTN(n=26)/DTN+ Bev(n=24)	bpCR, Safety	NR

**Legend and footnotes:** Year: year of registration; D= docetaxel; AC= doxorubicin+ cyclophosphamide; Bev= Bevacizumab; X= capecitabine; G= gemcitabine; EC= epirubicin+ cyclophosphamide; F= 5-fluorouracil; →: followed by; wP= weekly paclitaxel; nP= nab-paclitaxel; dd= dose dense; Carbo= carboplatin; T= trastuzumab; N= non-pegylated liposomal doxorubicin; Surgery: method of surgery, lumpectomy or mastectomy; NR=not reported.

**Table 2:** Biomarkers powered to predict Bev efficacy significantly at Baseline.

Molecular characteristic	Population	Outcome
High serum VEGF-A 35	HER2-/HR+BC	pCR
High serum VEGF-C 35	TNBC	pCR
Amplifications of VEGFA and TMEM100(an ALK1 receptor signaling‐dependent gene essential for vasculogenesis 36	HER2- BC	pCR
A set of five small RNAs associated with the regulation of VEGF pathway 18	HER2-/HR+ BC	pCR
Soluble carbonic anhydrase IX (sCAIX), upregulated in hypoxic conditions 37	HER2- BC	pCR, DFS
A small hypoxia signature 38	HER2- BC	pCR
BRCA1/2 mutations 39	TNBC	pCR, DFS
High proliferation, high p53 mutation and low IE (estrogen signaling) signatures 40	TNBC	pCR
Immune phenotype 41, 42	HER2-/ER+BC	pCR

**Table 3:** AEs related to Bev

AE (Grade 3-4)	Num	Pts	Rate	OR	I^2^
Hypertension**	7	4667	3.3%	4.41(1.75,11.10)	65.95%
Hand foot syndrome**	6	4592	4.9%	1.54(1.17,2.03)	0
Fatigue	5	2546	9.7%	1.18(0.91,1.54)	0
Diarrhea	5	2676	4.7%	0.76(0.53,1.10)	10.75%
Nausea	5	2676	4.6%	1.02(0.71,1.46)	0
Vomiting	5	2546	3.3%	0.81(0.52,1.28)	48.16%
Thromboembolic events	5	3820	2.4%	1.23(0.79,1.92)	46.37%
Neutropenia*	4	4274	53.3%	1.18(1.03,1.36)	0
Febrile neutropenia**	4	3612	10.1%	1.91(1.53,2.39)	0
Mucositis**	3	3540	6.4%	4.77(2.27,10.06)	56.12%
Headache**	3	1338	2.6%	4.83(1.98,11.80)	0
Dyspnea	3	1471	1.5%	2.22(0.87,5.65)	0
Surgical complications**	3	1927	18.4%	1.49(1.18,1.88)	48.96%

**Legend and footnotes:** Num= Number of included studies; Pts=Number of patients; Rate=Overall occurrence rate in all included patients; *: p<0.05; **: p<0.01.
