# Supplementary material for: Bevacizumab Plays a double-edged role in Neoadjuvant Therapy for Non-metastatic Breast Cancer: A Systemic Review and Meta-Analysis
Source: J Cancer. 2021 Mar 5;12(9):2643–53. doi: 10.7150/jca.53303 (PMC8040714; doi:10.7150/jca.53303)
Supplement: Supplementary file 1 — Supplementary appendices, figures and table. [file jcav12p2643s1.pdf]

Appendix A

Search Strategy

((((((((cancer[Title/Abstract]) OR tumor[Title/Abstract]) OR carcinoma[Title/Abstract]) OR neoplasm[Title/Abstract])) AND ((breast[Title/Abstract]) OR mammary[Title/Abstract]))) AND ((bevacizumab[Title/Abstract]) OR avastin[Title/Abstract])) AND ((neoadjuvant[Title/Abstract]) OR preoperative[Title/Abstract])) AND ((randomized controlled trial[Publication Type] OR randomized[Title/Abstract] OR placebo[Title/Abstract])).

Note: We used same search strategy in all three databases.

Appendix B

AEs included into meta-analysis

	study	Bev size	Bev event	Control size	Control event	Pts	rate
**AE1-Hypertension**							
Hypertension	TBCRC 002	50	4	25	0		
Hypertension	NSABP B40	595	65	596	5		
Arterial hypertension	GaperQuinto	951	25	965	4		
Hypertension	SWOG S0800	95	7	110	3		
Hypertension	CALGB 40603	216	24	217	2		
Hypertension	ARTemis	384	8	391	6		
Hypertension	AVATAXHER	47	0	25	1		
Total		2338	133	2329	21	4667	3.3%
**AE2-Hand foot symdrome**							
Hand-foot sydrome	NSABP B-40	595	65	596	42		
Hand-foot sydrome	GaperQuinto	951	52	965	33		
Hand-foot sydrome	SWOG S0800	95	1	110	2		
Peripheral neuropathy	CALGB 40603	216	10	217	9		
Peripheral neuropathy	ARTemis	384	6	391	4		
Peripheral sensory neuropathy	AVATAXHER	47	0	25	0		
Total		2288	134	2304	90	4592	4.9%
**AE3-Fatigue**							
Fatigue	TBCRC 002	50	1	25	0		
Fatigue	NSABP B-40	595	65	596	59		
Fatigue	CALGB 40603	216	25	217	22		
Fatigue	ARTemis	384	39	391	32		
Asthenia	AVATAXHER	47	3	25	0		
Total		1292	133	1254	113	2546	9.7%
**AE4-Diarrhea**							
Diarrhea	NSABP B-40	595	30	596	47		
Diarrhea	SWOG S0800	95	3	110	2		
Diarrhea	CALGB 40603	216	6	217	2		
Diarrhoea	ARTemis	384	16	391	20		
Diarrhoea	AVATAXHER	47	0	25	0		

Total		1337	55	1339	71	2676	4.7%
**AE5-Nausea**							
Nausea	NSABP B-40	595	30	596	29		
Nausea	SWOG S0800	95	6	110	9		
Nausea	CALGB 40603	216	13	217	7		
Nausea	ARTemis	384	13	391	17		
Nausea	AVATAXHER	47	0	25	0		
Total		1337	62	1339	62	2676	4.6%
**AE6-Vomiting**							
Nausea/Vomiting	TBCRC 002	50	0	25	0		
Vomiting	NSABP B-40	595	18	596	35		
Vomiting	CALGB 40603	216	6	217	4		
Vomiting	ARTemis	384	14	391	7		
Vomiting	AVATAXHER	47	0	25	0		
Total		1292	38	1254	46	2546	3.3%
**AE7-Thromboembolic events**							
Hemorrhagic and thrombotic event	TBCRC 002	50	3	25	0		
Thrombosis,thrombus,or embolism	NSABP B-40	595	11	596	17		
Thromboembolic events	GaperQuinto	951	26	965	18		
Thromboembolic events	SWOG S0800	95	1	110	2		
Thromboembolic events (SAE)	CALGB 40603	216	10	217	2		
Total		1907	51	1913	39	3820	2.4%
**AE-8 Neutropenia**	study						
Neutropenia	NSABP B-40	595	137	596	131		
Neutropenia	GaperQuinto	936	763	939	747		
Neutropenia	CALGB 40603	216	102	217	86		
Neutropenia	ARTemis	384	168	391	146		
Total		2131	1170	2143	1110	4274	53.3%
**AE9-Febrile neutropenia**							
Febrile neutropenia	NSABP B-40	595	65	596	41		
Febrile neutropenia,any grade	GaperQuinto	951	130	965	69		
Febrile neutropenia	CALGB 40603	216	36	217	20		
Febrile neutropenia	AVATAXHER	47	3	25	1		

Total		1809	234	1803	131	3612	10.1%
**AE10-Mucositis**							
Mucositis	NSABP B-40	595	29	596	6		
Mucositis	GaperQuinto	951	157	965	26		
Mucositis	CALGB 40603	216	4	217	3		
Total		1762	190	1778	35	3540	6.4%
**AE11-Headache**							
Headache	TBCRC 002	50	0	25	0		
Headache	NSABP B-40	595	29	596	5		
Headache	AVATAXHER	47	1	25	0		
Total		692	30	646	5	1338	2.6%
**AE12-Dyspnea**							
Dyspnea	TBCRC 002	50	0	25	0		
Dyspnea	NSABP B-40	595	11	596	5		
Dyspnea	SWOG S0800	95	3	110	1		
Total		740	14	731	6	1471	1.5%
**Surgical complication, any grade**							
wound healing complications	TORI B-02	30	10	32	2		
Surgical complications	NSABP B-40	560	141	562	106		
Surgical complications	Gaper Quinto	394	58	349	38		
Total		984	209	943	146	1927	18.4%

Supplementary figures and tables

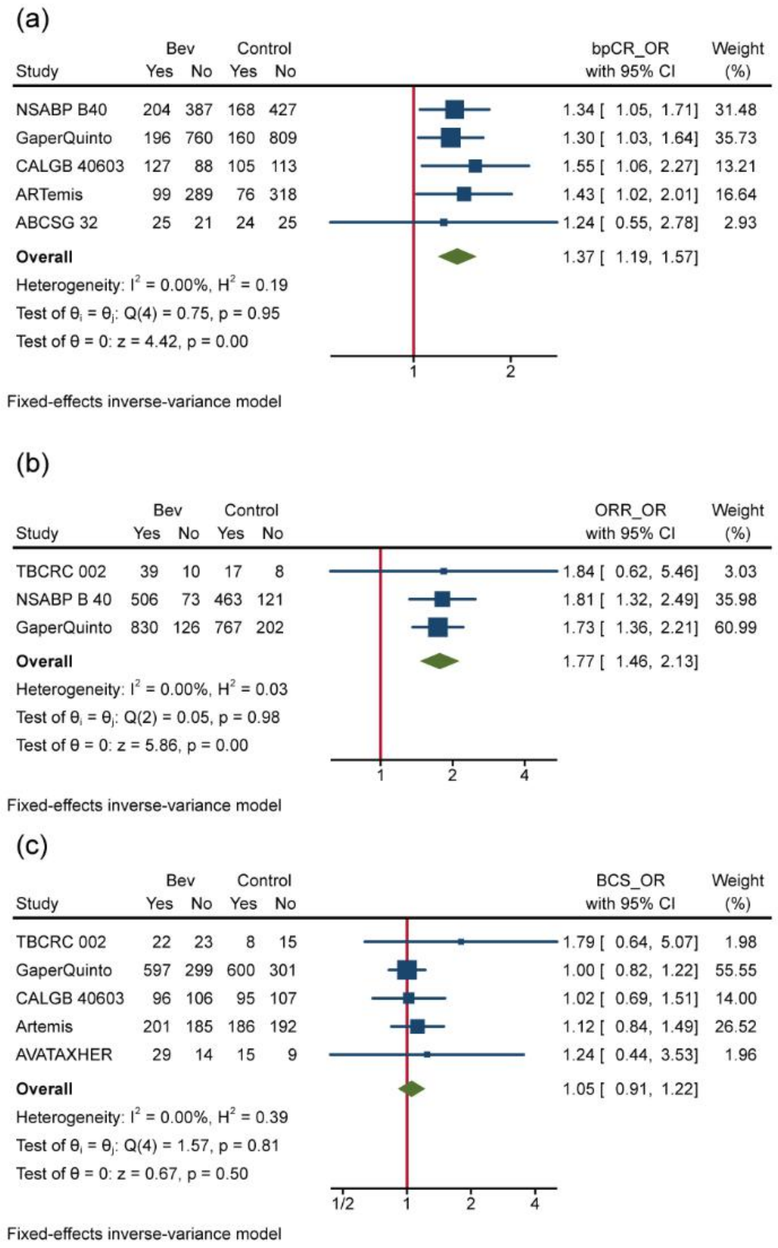

Figure S1. Efficacy of Bev Adding to NAT. (a) Pooled OR for bpCR. (b) Pooled HR for ORR. (C) Pooled HR for BCS.

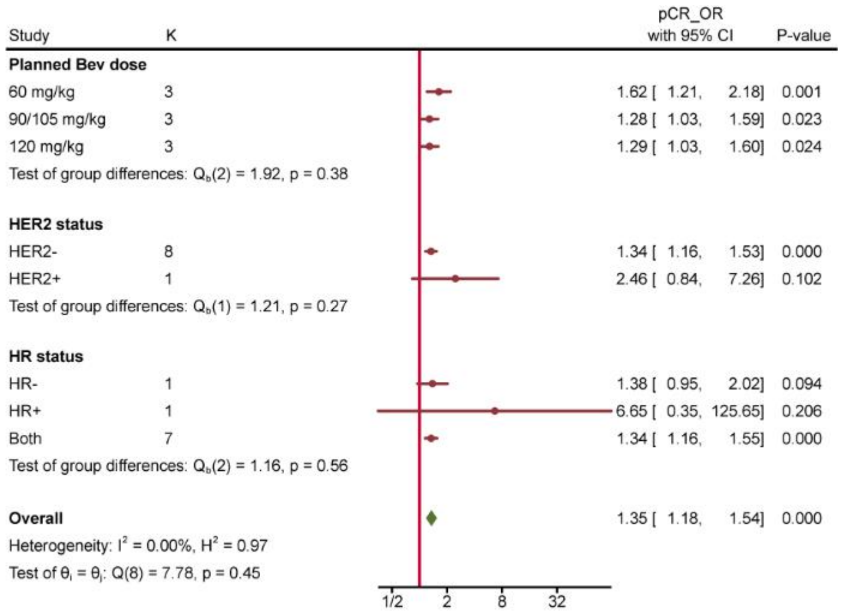

Figure S2. Subgroup Analysis. Comparing pCR between different subgroups.

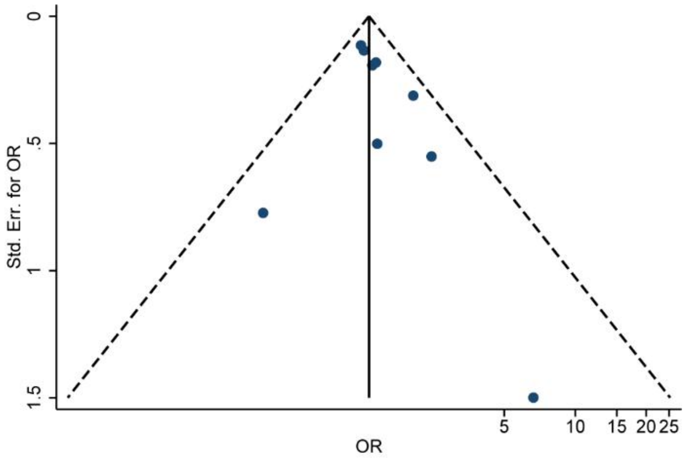

Figure S3. Funnel plot with pseudo 95% confidence limits

Table S1

β values calculated by nonlinear mixed model

	Num	group	B(slope)	SE
pCR~DFS	6	all	0.0878	46.8789
pCR~DFS	5	Bev pre-surgery only	0.1096	1.1187
pCR~OS	5	all	0.0703	4.1188
pCR~OS	4	Bev pre-surgery only	0.1053	2.5227
DFS~OS	5	all	1.5947	1.0134

Legend and footnotes: Num= Number of included studies; SE= Standard error.
